# Multidimensional effects of biologically synthesized silver nanoparticles in *Helicobacter pylori*, *Helicobacter felis*, and human lung (L132) and lung carcinoma A549 cells

**DOI:** 10.1186/s11671-015-0747-0

**Published:** 2015-02-05

**Authors:** Sangiliyandi Gurunathan, Jae-Kyo Jeong, Jae Woong Han, Xi-Feng Zhang, Jung Hyun Park, Jin-Hoi Kim

**Affiliations:** Department of Animal Biotechnology, Konkuk University, 1 Hwayang-Dong, Gwanjgin-gu, 143-701 Seoul South Korea; GS Institute of Bio and Nanotechnology, Coimbatore, Tamilnadu India

**Keywords:** Silver nanoparticles, *Helicobacter pylori*, *Helicobacter felis*, Human lung cells, Lung carcinoma cells, Cell viability, ROS, Mitochondrial membrane potential

## Abstract

Silver nanoparticles (AgNPs) are prominent group of nanomaterials and are recognized for their diverse applications in various health sectors. This study aimed to synthesize the AgNPs using the leaf extract of *Artemisia princeps* as a bio-reductant. Furthermore, we evaluated the multidimensional effect of the biologically synthesized AgNPs in *Helicobacter pylori*, *Helicobacter felis*, and human lung (L132) and lung carcinoma (A549) cells. UV-visible (UV–vis) spectroscopy confirmed the synthesis of AgNPs. X-ray diffraction (XRD) indicated that the AgNPs are specifically indexed to a crystal structure. The results from Fourier transform infrared spectroscopy (FTIR) indicate that biomolecules are involved in the synthesis and stabilization of AgNPs. Dynamic light scattering (DLS) studies showed the average size distribution of the particle between 10 and 40 nm, and transmission electron microscopy (TEM) confirmed that the AgNPs were significantly well separated and spherical with an average size of 20 nm. AgNPs caused dose-dependent decrease in cell viability and biofilm formation and increase in reactive oxygen species (ROS) generation and DNA fragmentation in *H. pylori* and *H. felis*. Furthermore, AgNPs induced mitochondrial-mediated apoptosis in A549 cells; conversely, AgNPs had no significant effects on L132 cells. The results from this study suggest that AgNPs could cause cell-specific apoptosis in mammalian cells. Our findings demonstrate that this environmentally friendly method for the synthesis of AgNPs and that the prepared AgNPs have multidimensional effects such as anti-bacterial and anti-biofilm activity against *H. pylori* and *H. felis* and also cytotoxic effects against human cancer cells. This report describes comprehensively the effects of AgNPs on bacteria and mammalian cells. We believe that biologically synthesized AgNPs will open a new avenue towards various biotechnological and biomedical applications in the near future.

## Background

Nanomaterials often have novel and size-related physico-chemical properties that differ significantly from their larger counterparts. Therefore, the growing interest in the field has driven the production of numerous nanomaterials with outstanding optical, magnetic, catalytic, and electrical properties [1,2]. Silver nanoparticles (AgNPs) have become increasingly popular and have been used in various applications such as antibiotic agents in textiles and wound dressings and in biomedical devices; furthermore, they are one of the most commonly used engineered nanoparticles in commercialized products [3,4]. Since AgNPs have widespread applications, academia and industry have paid more attention to the production of AgNPs than to their uses [5].

Among several methods, chemical methods provide an easy way to synthesize AgNPs in solution, and they are a commonly used method for the production of AgNPs [5]. In contrast, physical methods appear to produce a low yield. Chemical methods, on the other hand, are more complex in that they require three main components, including metal precursors, reducing agents, and stabilizing/capping agents. Furthermore, chemical methods use various toxic materials including hydrazine, citrate, borohydride, or other organic compounds (*e.g.*, reducing agents); all these agents can be toxic to living organisms including humans. Capping agents are playing an important role for the stabilization of nanoparticles, for example, capped AgNPs exhibit better antibacterial activity than uncapped AgNPs do [6,7]. Biological methods seem to be valuable for the preparation of AgNPs with controlled size and shape of the nanoparticles [8-13]. Given that conventional physical methods have low yields and chemical methods are toxic and consume a lot of energy, the development of environmentally friendly approaches has become the more preferred trend for the field of nanobiotechnology. Biologically prepared nanomaterials are extremely valuable because nanoparticles are easily soluble and stable [14]. In addition, during the biological synthesis of AgNPs, the reducing agent and stabilizer are replaced by molecules produced by living organisms. These molecular compounds can be sourced from various living organisms such as bacteria, fungi, yeasts, algae, or plants [15]. Biomolecules can be attached to various types of surfaces via diffusion, adsorption/absorption, covalent cross-linking, and affinity interaction [16].

Recently, numerous microorganisms have been reported to synthesize AgNPs, including bacteria like *Pseudomonas stutzeri* AG259 [17], *Bacillus licheniformis* [10], *Brevibacterium casei* [18], *Escherichia coli* [9], and *Shewanella oneidensis* [19] and fungi like *Fusarium oxysporum* [20], *Trichoderma viride* [21], and *Ganoderma neo-japonicum* [21]. Extracellular synthesis of various types of nanoparticles was performed using plants, including geranium leaves [22] and lemongrass [23], via the reduction of aqueous AgNO_3_ and AuCl_4_, respectively. Previous studies suggest that leaf and other parts of plant extracts from various plants, such as *Azadirachta indica* [24], *Aloe vera* [25], *Bryophyllum* sp. [26], *Gliricidia sepium,* Alfalfa sprouts [27,28], aqueous stem extract of banana [29], and *Allophylus cobbe* [8], have also been explored for the synthesis of AgNPs. Compared to other reducing agents derived from microorganisms, the reduction of the Ag^+^ ions with the extracts of plants occurs quickly [22]. Furthermore, biological methods seem to have less time required for complete reduction and be stable and readily available in solution at high densities [13]. Similarly, shape and size, the rate of reduction of metal ions is faster, and more stable metal nanoparticles are formed using leaf extracts compared to using microorganisms [28,30].

The green juice of *Artemisia princeps* used to treat skin injuries and gastrointestinal disorders [31,32]. Yun et al. [33] have identified 16 water-soluble phenolic compounds in the leaf water extract of *A. princeps*, and its extract contains 192 volatile chemicals [31]. Therefore, this plant extract can be used as a reducing and stabilizing agent for the synthesis of AgNPs.

Infections caused by multidrug-resistant bacteria lead to major public health issues, such as morbidity, mortality, cost of health care, and the need for implementation of infection control measures [34]. Parsonnet et al. [35] reported that bacteria have been linked to cancer by the induction of chronic inflammation and the production of carcinogenic bacterial metabolites. A pertinent example of the inflammatory mechanism of carcinogenesis is the *Helicobacter pylori* infection. *H. pylori* are known to cause infection in the stomach and are found in about two thirds of the world’s population. *H. pylori* exist and are adherent to the epithelium of stomach. Non-pylori gastric *Helicobacter* organisms cause chronic gastritis and inflammation in humans [36]*.* On the other hand, *Fusobacterium nucleatum* promotes colorectal carcinogenesis and intestinal tumorigenesis and modulates the tumor-immune microenvironment [37,38].

Recent surveys suggest that lung cancer accounts for 23% of all cancer-related mortality, outnumbering the total mortality of breast, colon, and prostate cancers combined [39,40]. To address the effect of AgNPs, several studies have reported the impact of AgNPs in various cell lines, such as BRL4A rat liver cells [41], PC-12 neuroendocrine cells [42], germ line stem cells [43], rat alveolar macrophages [44], and a human lung carcinoma cell line, A549 [45]. Recent studies reported that biologically prepared AgNPs have been used for antibacterial and antifungal [46-48]. The results from previous studies suggest that the generation of reactive oxygen species (ROS) is an important and general mechanism of nanoparticle-mediated cytotoxicity through DNA damage, apoptosis, and necrosis [44,49-53]. Although various studies have addressed the effect of AgNPs in various cell lines, there has been no study on the multiple functions of biologically prepared AgNPs using *A. princeps* on bacteria causing carcinogenesis and human cancer cells. Therefore, this study was aimed to investigate the following objectives. Firstly, we aimed to develop an easy, consistent, cost-effective, and green approach to the synthesis of colloidal AgNPs using leaf extract of *A. princeps.* Secondly, we evaluated the antibacterial and anti-biofilm activity of AgNPs against *H. pylori* and non-pylori *Helicobacter felis.* Finally, we assessed the cell-specific cytotoxic effects of AgNPs in normal lung and lung cancer cells.

## Methods

### Bacterial strains and reagents

All culture media and chemicals were purchased from Sigma-Aldrich (St. Louis, MO, USA) unless otherwise stated. The strains of *H. pylori* GS-13 and *H. felis* GS-14 used in the present study were obtained from our culture collection. All strains were maintained at −80°C in Brucella agar (BA) (Sigma, Cream Ridge, NJ, USA) supplemented with 2% fetal calf serum (FCS). Penicillin-streptomycin solution, trypsin-EDTA solution, RPMI 1640 medium, and 1% antibiotic-antimycotic solution were obtained from Life Technologies/Gibco (Grand Island, NY, USA). Silver nitrate, fetal bovine serum (FBS), and the *in vitro* toxicology assay kit were purchased from Sigma-Aldrich (St. Louis, MO, USA).

### Synthesis and characterization of AgNPs

*A. princeps* leaves were collected from plants growing in the Jeju Island, South Korea, and stored at 4°C until needed. The leaf extract was prepared according to method described earlier [54]. Briefly, the filtered extract was used for the synthesis of AgNPs by adding 10 mL (1 mg/mL) to 100 mL of 1 mM AgNO_3_ in an aqueous solution at room temperature. The bio-reduction of the AgNO_3_ was monitored spectrophotometrically between 300 and 600 nm. The synthesized particles were characterized according to methods described previously [9]. The size distribution of the dispersed particles was measured using a Zetasizer Nano ZS90 (Malvern Instruments Ltd., Malvern, UK). X-ray diffraction (XRD) analyses were carried out on an X-ray diffractometer (Bruker D8 DISCOVER; Bruker AXS GmBH, Karlsruhe, Germany). The high-resolution XRD patterns were measured at 3 Kw with a Cu target using a scintillation counter (*λ* = 1.5406 °A) at 40 kV and 40 mA and were recorded in the range of 2*θ* = 5°–80°. Further characterization of changes in the surface and surface composition was performed by Fourier transform infrared spectroscopy (PerkinElmer Spectroscopy GX, PerkinElmer, Waltham, MA, USA). Transmission electron microscopy (TEM), using a JEM-1200EX microscope, was performed to determine the size and morphology of AgNPs. TEM images of AgNPs were obtained at an accelerating voltage of 300 kV.

### Determination of minimum inhibitory concentrations of AgNPs and *in vitro* killing assay

Minimal inhibitory concentration (MIC) of *H. pylori* and *H. felis* was determined as described previously [8]. To determine the MICs of AgNPs, *H. pylori* and *H. felis* were then exposed to different concentrations of AgNPs. Growth was monitored using a microtiter ELISA reader (EMax, Molecular Devices, Sunnyvale, CA, USA) by monitoring the absorbance at 600 nm. The MIC of AgNPs was defined as the lowest concentration that inhibited the visible growth of bacteria. The *in vitro* killing assay was performed as described previously [8].

### Determination of biofilm activity using the tissue culture plate method

Inhibition of biofilm was determined as described earlier with suitable modifications [8,55]. Briefly, the cells were grown in Brucella broth supplemented with 2% FCS and individual wells of sterile, 96-well flat-bottom polystyrene tissue culture plates (TCPs) were filled with 180 μL of a single bacterial species (1 × 10^6^/mL). The cell culture plates were then incubated with AgNPs for 24 h at 37°C. After incubation, the media were removed, and the wells were washed three times with 200 μL sterile distilled water to remove non-adherent bacteria. The crystal violet solutions in water were added for 45 min. The wells were then washed five times with 300 μL of sterile distilled water to remove excess stain. The absorbance of each well was measured at 595 nm using a microtiter ELISA reader. The percent inhibition of biofilm activity was calculated as described earlier [8,55].

### Measurement of ROS generation in bacteria

ROS was determined according to the manufacturer’s instructions and according to previous publications [8,49,56]. All test strains were grown in BB. Cell suspensions were incubated with AgNPs at 37°C on a rotary shaker for 12 h. Aliquots were then removed and spun in a microfuge, and the absorption of the supernatant was measured at 450 nm.

### Cell culture

Human lung cancer A549 cells and normal human lung L-132 cells were obtained from the Korean Cell Bank (Seoul, Korea) and cultured in RPMI 1640 medium supplemented with 10% FBS and 100 U/mL penicillin-streptomycin at 5% CO_2_ and 37°C. At 75% confluence, the cells were harvested using 0.25% trypsin and subcultured in 75-cm^2^ flasks, 6-well plates, or 96-well plates. Cells were allowed to attach the surface for 24 h before treatment. The medium was replaced three times per week, and the cells were passaged at subconfluency.

### Cell viability and cytotoxicity assays

Cell viability was measured using a Cell Counting Kit-8 (CCK-8; Dojindo Laboratories, Kumamoto, Japan). Briefly, A549 and L132 cells were plated onto 96-well flat-bottom culture plates with various concentrations of AgNPs. All cultures were incubated for 24 h at 37°C (5% CO_2_ in a humidified incubator). CCK-8 solution (10 μL) was added to each well, and the plate was incubated for another 2 h at 37°C. Absorbance was measured at 450 nm with a microplate reader (Multiskan FC; Thermo Fisher Scientific Inc., Waltham, MA, USA). Cytotoxicity was assessed using supernatants from the medium in lactate dehydrogenase (LDH) assays. An LDH Cytotoxicity Detection kit (Takara Bio Inc., Tokyo, Japan) was used according to the manufacturer’s protocol, and the absorbance was measured at 490 nm using a microplate reader.

### ROS (H_2_-DCFH-DA) assay

Human lung normal L132 cells and A549 human lung epithelial adenocarcinoma cells were cultured in minimum essential medium (Hyclone Laboratories, Logan, UT, USA) containing 10 μM H_2_-DCFDA in a humidified incubator at 37°C for 30 min. Cells were washed in PBS (pH 7.4) and lysed in lysis buffer (25 mM HEPES [pH 7.4], 100 mM NaCl, 1 mM EDTA, 5 mM MgCl_2_, and 0.1 mM DTT supplemented with a protease inhibitor cocktail). Cells were cultured on coverslips in a 4-well plate. Cells were incubated in DMEM containing 10 μM H_2_-DCFDA at 37°C for 30 min. Cells were washed in PBS, mounted with Vectashield fluorescent medium, and viewed with a fluorescence microscope.

### JC-1 assay

The change in mitochondrial transmembrane potential was evaluated using the cationic fluorescent indicator JC-1 (Molecular Probes, Eugene, OR, USA). J-aggregates of intact mitochondria were fluorescent red with an emission at 583 nm, and J-monomers in the cytoplasm were fluorescent green with emission at 525 nm and an excitation wavelength of 488 nm. A549 and L132 cells were incubated in RPMI containing 10 μM JC-1 at 37°C for 15 min, washed with PBS, and transferred to a clear 96-well plate. Cells were also cultured on cover slips, incubated in DMEM containing 10 μM JC-1 at 37°C for 15 min, and then washed with PBS. Finally, cells were mounted using Vectashield fluorescent medium and visualized with fluorescence microscopy.

### Statistical analyses

All assays were carried out in triplicate and the experiments were repeated at least three times. The results are presented as means ± SD. All experimental data were compared using Student’s *t*-test. A *p* value less than 0.05 was considered statistically significant.

## Results and discussion

### Synthesis and characterization of AgNPs using leaf extract

**Figure 1:**
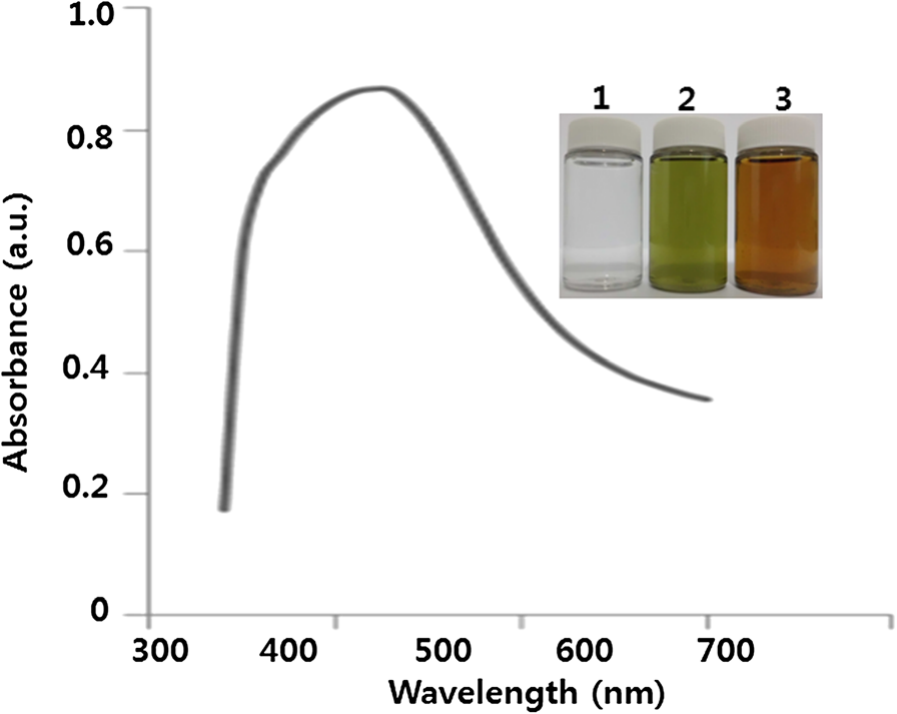
**Synthesis and characterization of AgNPs using leaf extract of** ***A. princeps.*** The inset shows tubes containing samples of silver nitrate (AgNO_3_) after exposure to 60 min (1), leaf extract (2), and AgNO_3_ plus leaf extract (3). The color of the solution turned from pale yellow to brown after 15 min of incubation, indicating the formation of silver nanoparticles. The absorption spectrum of AgNPs synthesized by leaf extract exhibited a strong broad peak at 410 nm, and observation of such a band is assigned to surface plasmon resonance of the particles.

Synthesis of AgNPs using leaf extract was performed according to a previously described method [8]. The leaf extract of *A. princeps* was used as both a reducing and stabilizing agent. In a typical reaction procedure, 10 mL of *A. princeps* leaf extract was added to 100 mL of 1 mM aqueous AgNO_3_ solution under magnetic stirring at room temperature for 60 min. The color change was observed by visual observation in the tube, which contains AgNO_3_ solution with leaf extract. The mixture of silver nitrate and leaf extract changed rapidly from green to a brown suspended mixture after 15 min, whereas silver nitrate without leaf extract exhibited no color change (Figure 1 inset). The synthesis of AgNPs using the leaf extract was confirmed by the color change. To monitor the synthesis and stability of AgNPs, the absorption spectra of the AgNPs were observed using UV-visible spectroscopy. The color of the solutions changed from pale yellow to yellowish brown to deep brown depending on the extract concentration; this indicates that AgNP formation occurs due to the excitation of surface plasmon vibration (SPR) of the particles. The typical SPR of AgNPs was observed between 410 and 420 nm (Figure 1).

### XRD analysis of AgNPs

**Figure 2:**
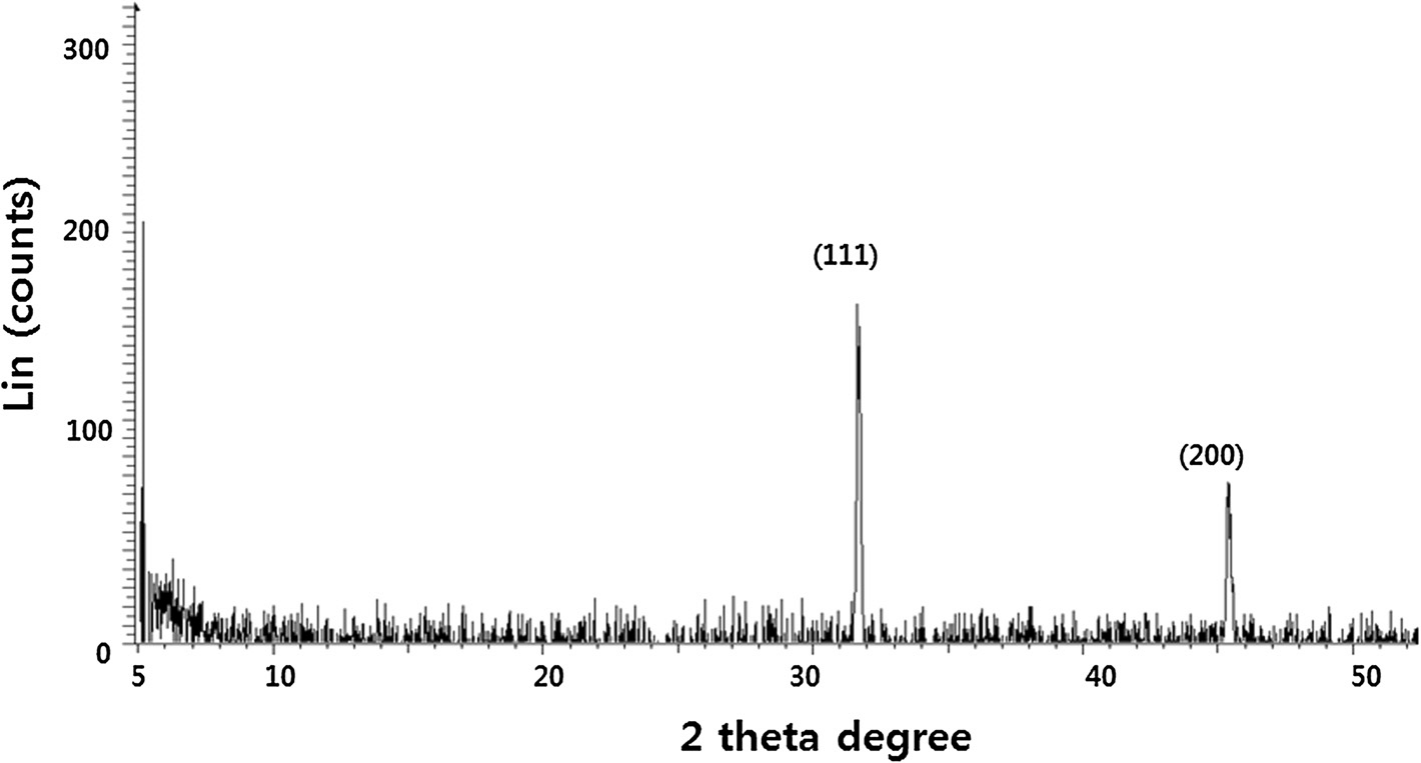
**XRD pattern of AgNPs.** A representative X-ray diffraction (XRD) pattern of silver nanoparticles formed after reaction of leaf extract with 1 mM of silver nitrate (AgNO_3_) for 60 min at 60°C. The XRD pattern shows two intense peaks in the whole spectrum of 2*θ* values ranging from 20 to 70. The intense peaks were observed at 2*θ* values of 31.9 and 45.31 corresponding to (111) and (200) planes for silver, respectively.

The crystalline nature of the AgNPs was confirmed using XRD, and the XRD pattern revealed Bragg’s reflections that represent the face-centered cubic structure of silver. Figure 2 shows the XRD patterns of dried AgNPs synthesized with *A. princeps* leaf extract. The XRD patterns indicated that the structure of AgNPs is face-centered cubic (fcc) [8,57]. The sharp XRD peaks at 2*θ* of 31.9 and 45.3 are attributed to the (111) and (200) crystallographic planes. The two diffraction peaks could be indexed as (111), (200), (220), (311), and (222) planes of fcc silver (JCPDS, fileno.04-0783) [24]. A comparison made between our XRD spectrum and the standard confirmed that our silver particles exhibited Bragg’s reflections of silver. Hence, the XRD results clearly show that the AgNPs are crystalline. Interestingly, the XRD pattern shows no impurities as reported earlier [58]. From the XRD data and the use of the Debye-Scherer equation, the average particle size was 20 ± 3.5 nm. Remarkably, the absence of peaks from other phases suggests that nanoparticles with single-phase Ag with cubic structure were synthesized. Awwad et al. [58] reported that using carob leaf extract as a reducing and stabilizing agent produced 5- to 50-nm sizes of AgNPs. Recently, Mukherjee et al. [59] reported that the AgNPs synthesized with leaf extract from *Olax scandens* were mostly monodispersed and spherical (20 to 60 nm) along with very few larger particles (approximately 90 nm). Our studies suggest that *A. princeps* shows a significantly uniform distribution of particles with an average size of 20 nm.

### FTIR spectra of AgNPs

**Figure 3:**
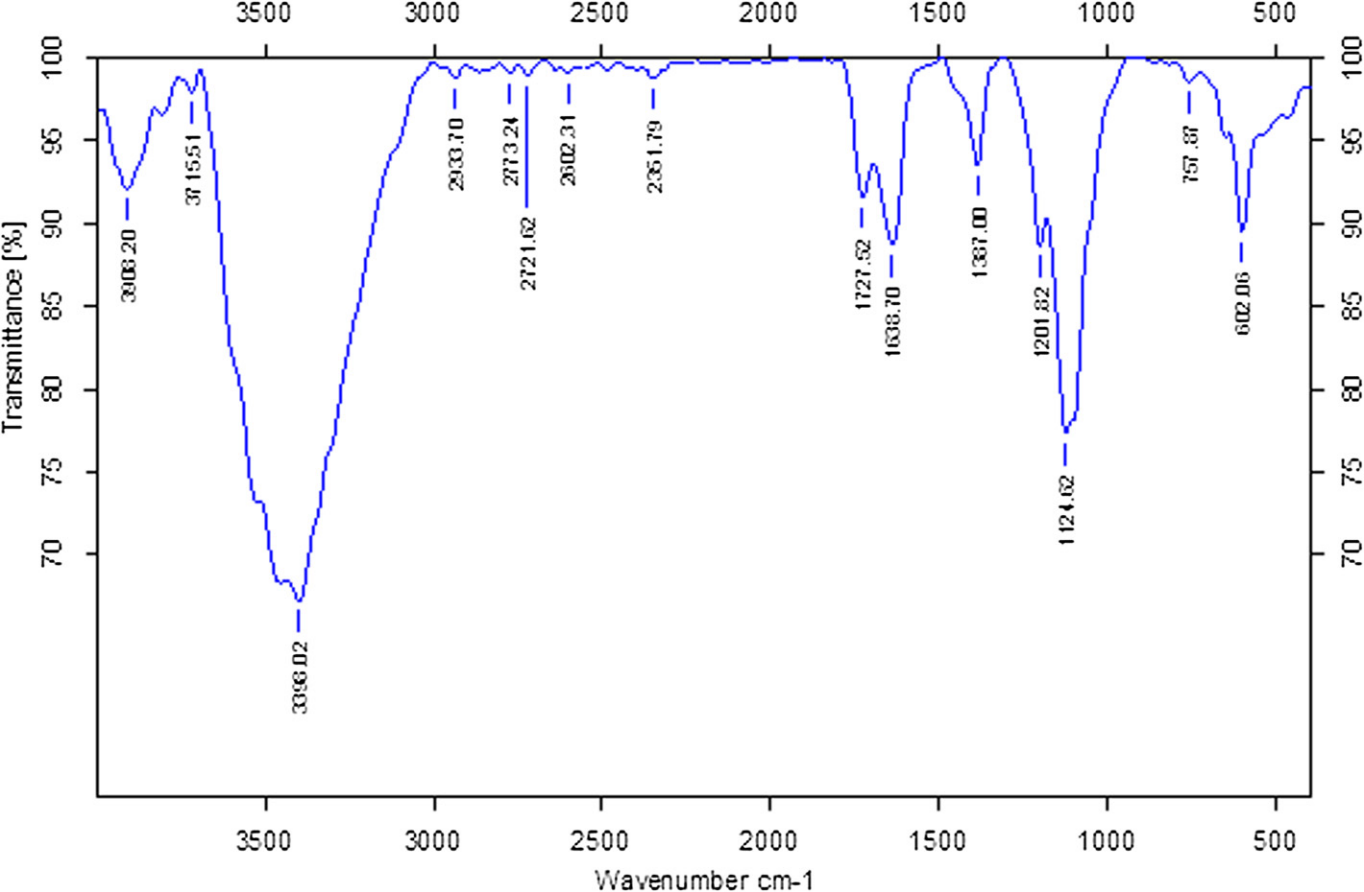
**FT-IR spectrum of silver nanoparticles synthesized by treating AgNO**_**3**_ **with leaf extract.**

To identify the possible biomolecules involved in the reduction of the Ag^+^ by the extract of leaf broth, we used Fourier transform infrared spectroscopy (FTIR) measurements [22]. The silver nitrate solution after completing the reduction of Ag^+^ ions and the formation of AgNPs was centrifuged at 10,000 rpm for 15 min to isolate the AgNPs from free proteins [22]. The FTIR spectrum of AgNPs exhibits peaks at 1,727 and 1,638 cm^−1^ that are attributed to ester CdO groups of chlorophyll [22,60]. It is well known that water-soluble fractions of *A. princeps* leaves contain large amounts of secondary metabolites; these secondary metabolites may favor the synthesis of nanoparticles as reducing agents [31]. Terpenoids possibly contribute to the reduction of the silver ions that, in the process, are oxidized to carbonyl groups, resulting in a band at 1,727 cm^−1^. During the formation of AgNPs, the peak corresponding to the amide I band at 1,638 cm^−1^ broadened, which indicates capping of the AgNPs by protein (Figure 3). The absorption peak at 1,638 cm^−1^ may be ascribed to the carbonyl stretch in proteins, while the peaks at 3,398 cm^−1^ represent the OH stretching in alcohols and phenolic compounds [61-63]. The strong intense peaks at 1,387 and 1,638 cm^−1^ correspond to C-N stretch vibrations, as well as to the amide I bands of proteins in the leaf extract. The absorption peak at 1,638 cm^−1^ is close to that reported for native proteins [64]. This suggests that proteins are interacting with biosynthesized nanoparticles, and their secondary structure were not affected during the reaction with Ag^+^ ions. Proteins are able to bind with silver or gold nanoparticles either through free amine groups or cysteine residues in the proteins [65]. A similar mechanism may be involved when the leaf extract from *A. princeps* caps the AgNPs, thereby stabilizing the particles. Similarly, several other researchers found a similar FTIR pattern of AgNPs using geranium leaf extract [22], *Ocimum sanctum* leaf extract [66,67], and *Camellia sinensis* [62]. The results obtained from FTIR spectroscopy suggest that the leaf extracts have the ability to reduce and stabilize the AgNPs. The present results agree with those reported previously for *Rhinacanthus nasutus* [68], *Thevetia peruviana* [69], latex of Jatrophacurcas [67], *O. sanctum* leaf extract [63,66], and *C. sinensis* [62].

### Dynamic light scattering analysis of AgNPs

**Figure 4:**
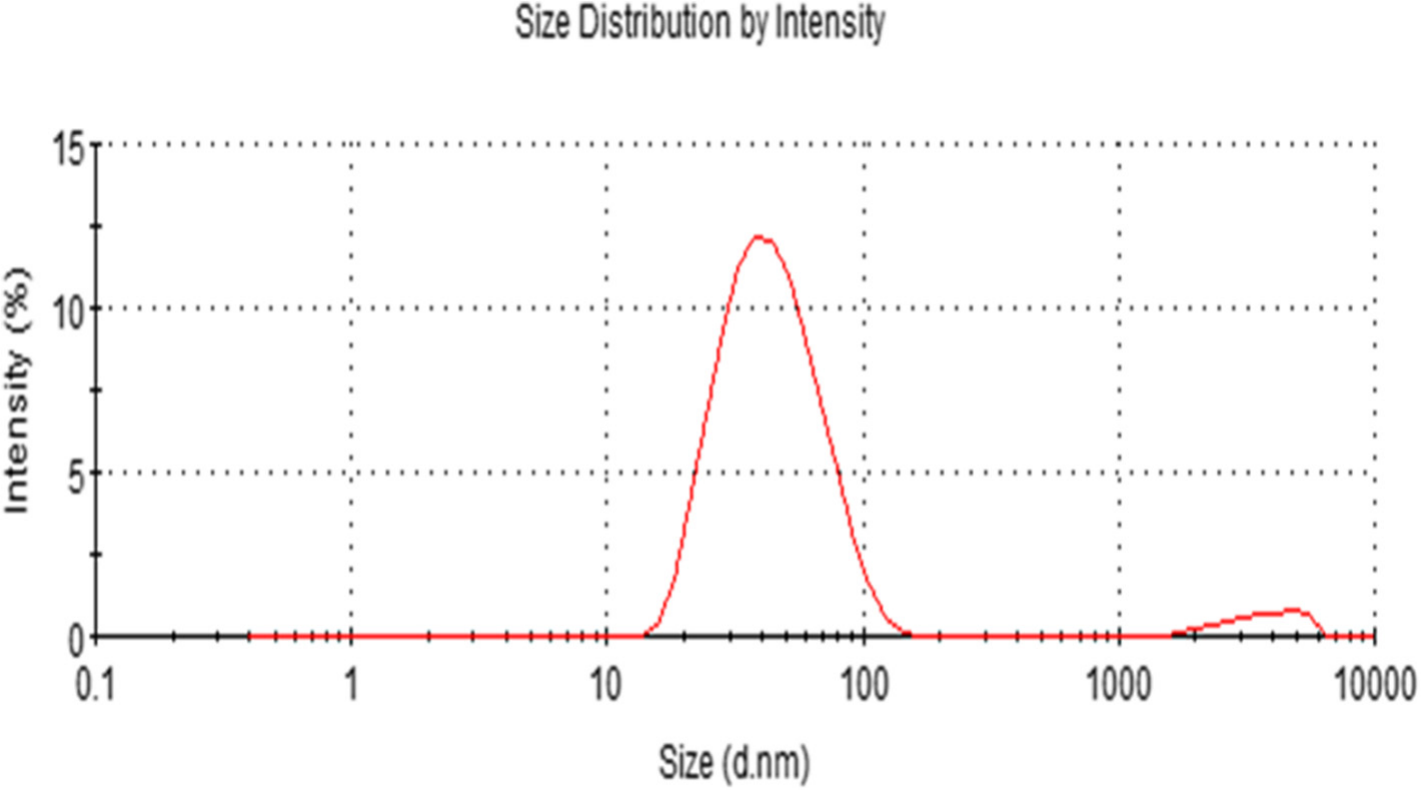
**Size distribution analysis by dynamic light scattering (DLS).** Silver nanoparticles were dispersed in deionized water and the particles were mixed thoroughly via sonication and vortexing, and samples were measured at 25 μg/mL.

Dynamic light scattering (DLS) measurement was used to determine the size of synthesized AgNPs in aqueous solution. Powers et al. [70] proposed DLS to be one of the most valuable and useful technique to evaluate particle size and size distribution of any nanomaterial in solution [71]. Specifically, particle size, size distribution, particle morphology, particle composition, surface area, surface chemistry, and particle reactivity in solution are important factors to assess the nanoparticle toxicity [72]. DLS characterizes the size of colloidal dispersions using the illumination of a particle or molecule suspension undergoing Brownian motion by a laser beam [72]. The present study used DLS, in conjunction with TEM, to evaluate the size distribution. The DLS pattern revealed that the synthesized AgNPs showed an average size of 40 ± 15 nm (Figure 4). From the figure, the particles obtained were monodisperse in the range 10 to 100 nm and had an average size of 40 nm. This size is larger than the TEM data, which is due to the hydrodynamic size of nanomaterials. Singhal et al. [63] reported that the AgNPs synthesized using *O. sanctum* leaf extract showed an average diameter of 22.38 nm. Umoren et al. [72] reported that the average size of the synthesized AgNPs using red apple fruit extract is around 150 nm. The sizes and shapes of metal nanoparticles are influenced by a number of factors, including pH, precursor concentration, reductant concentration, time of incubation, temperature, and the method of preparation [9].

### Size and surface analysis of AgNPs by TEM

**Figure 5:**
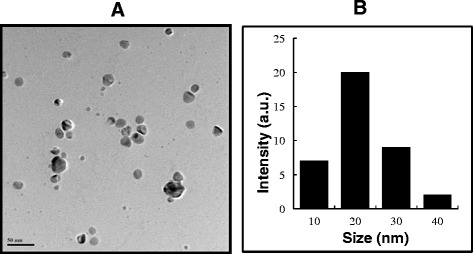
**Size and surface morphology of AgNPs analysis by TEM. (A)** Several fields were photographed and used to determine the diameter of silver nanoparticles (AgNPs) using TEM. The average range of observed diameter was 20 nm. **(B)** Histogram of Particle size from transmission electron microscopy images.

TEM is widely used to directly and accurately analyze the structural and size information of nanoparticles. TEM was used to characterize the nanoparticle sizes and morphologies [71]. Next, we examined the size and surface morphologies of synthesized AgNPs. TEM micrographs of AgNPs show distinct, uniform, and spherical nanoparticles that were widely separated. The average particle size was estimated from more than 300 particles and determined to be between 5 and 30 nm with an average size of 20 nm (Figure 5A). It was observed that AgNPs were evenly distributed in the analyzed sample (Figure 5B). Shankar et al. [22] reported that the size of the nanoparticles produced by geranium leaf extract from 16 to 40 nm. Mukherjee et al. [59] reported that synthesized AgNPs using *O. scandens* leaf extract shows almost monodispersed spherical nanoparticles (20 to 60 nm) along with very few bigger particles (approximately 90 nm). Interestingly, our data suggest that using *A. princeps* could produce smaller AgNPs, which are better for antimicrobial activity and anticancer activity. The image obtained from TEM study shows that the morphology of AgNPs is spherical, which is in agreement with the shape of the SPR band in the UV-visible (UV–vis) spectrum. The particle size could be controlled by varying the parameters such as temperature, pH, and concentration of AgNO_3_ [9].

### Determination of MIC of AgNPs against *H. pylori* and *H. felis*

In response to the overwhelming evidence linking *H. pylori* infection to human cancer, the International Agency for Research on Cancer listed *H. pylori* as a definite human oncogenic agent in 1994 [36,73-75]. Therefore, we were interested in finding out the MIC of AgNPs against both pylori and non-pylori strains, such as *H. pylori* and *H. felis*, respectively. The MIC of AgNPs was defined as the lowest concentration that completely inhibited visible growth of bacteria after incubation at 37°C for 24 h. In these studies, *H. pylori* and *H. felis* were used as a model bacteria for Gram negative to evaluate antibacterial activities of AgNPs. Both strains were incubated with the different concentration of AgNPs for 24 h in Brucella broth. The media without AgNPs were used as a control. The *H. pylori* and *H. felis* bacterial counts were significantly reduced by the treatment with AgNPs than control. The level of MIC of AgNPs was found to be 5.0 and 5.5 μg/mL to *H. pylori* and *H. felis*, respectively. The toxic effects of AgNPs depend on size, surface area, and surface functionalization which are major factors that influence bio-kinetics and toxicity in bacteria [76,77].

### Dose-dependent antibacterial effects of AgNPs

**Figure 6:**
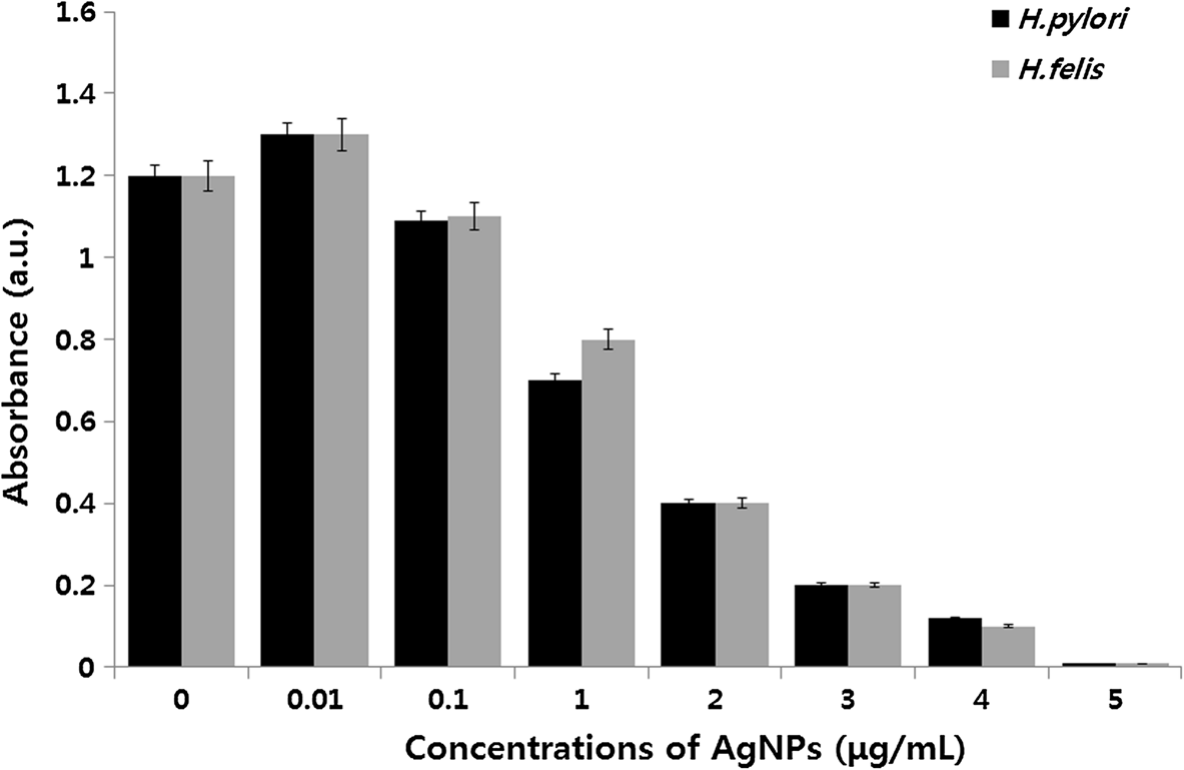
**Effect of AgNPs on cell survival of** ***H. pylori*** **and** ***H. felis.*** Dose-dependent effects of AgNPs on bacterial survival. All test strains were incubated in the presence of different concentrations of AgNPs. Bacterial survival was determined at 4 h by a CFU assay.

The antimicrobial effect of Ag^+^ ions has been well documented and it has been applied [78,79]. We are interested to investigate the potential of antibacterial activity of biologically synthesized AgNPs against *H. pylori* and *H. felis.* The dose-dependent antibacterial activity of AgNPs was determined using representative Gram-negative bacterial strains, *H. pylori*, and *H. felis*. Figure 6 shows the toxicity of biologically synthesized AgNPs (20 nm) at concentrations of 0.01 to 5 μg/mL to *H. pylori* and *H. felis.* Cell viability was reduced as the concentrations of the AgNPs increased [8]. For each bacterial strain, no growth was observed at their respective MIC values. Thus, these represent bactericidal concentrations for each specific bacterial strain. The plant extract-mediated AgNPs exhibited significant antimicrobial activity. For example, Li et al. [80] reported that 10 μg/mL (AgNPs) could completely inhibit the growth of 10^7^ CFUs/mL of *E. coli* in liquid MHB. Anthony et al. [81] reported that 10 μg/mL treatments of AgNPs with an average size of 40 nm decreased the cell viability completely in *Pseudomonas aeruginosa*. Kim et al. [76] investigated the antimicrobial activity of Ag nanoparticles against bacteria and yeast, and they found that *E. coli* shows more sensitivity at low concentration of Ag nanoparticles, whereas the effects of AgNPs on *Staphylococcus aureus* were mild. Our studies show that a promising inhibitory effect of AgNPs against tested strains was observed with concentration of 5 μg/mL. Previous studies showed that AgNPs are effective antimicrobial agents and mechanisms of toxicity attached with cell membrane and disturb their functions such as permeability and respiration [77,82-84]. Our results suggest that AgNPs synthesized using plant extract of *A. princeps* seem to be smaller in size, which may provide more bactericidal effects than larger particles.

### Time-dependent antibacterial activity of AgNPs

**Figure 7:**
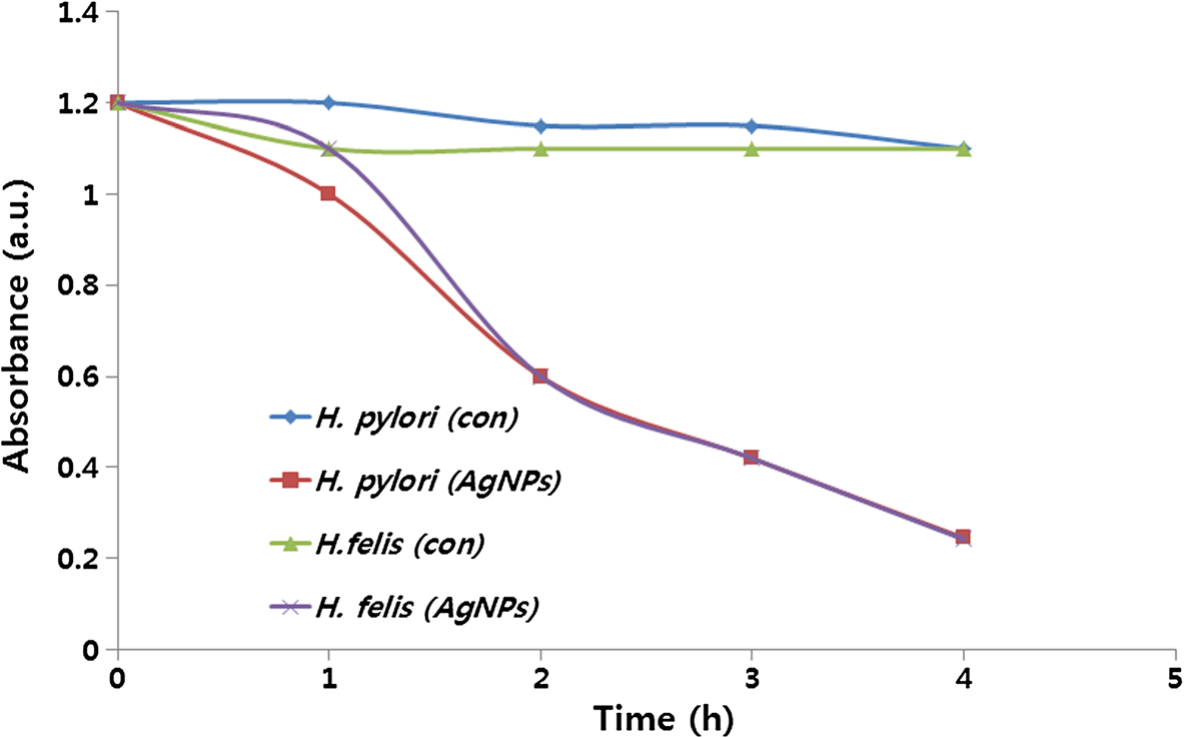
**Time-dependent effect of AgNPs on** ***H. pylori*** **and** ***H. felis.*** Time-dependent effects of AgNPs on bacterial survival. All test strains were incubated in the presence of different concentrations of AgNPs. Bacterial survival was determined at 4 h by a CFU assay.

The time-dependent antibacterial efficiency of AgNPs was determined in *H. pylori* and *H. felis*. The loss of viability of *H. pylori* and *H. felis* was counted at different time points such as 1, 2, 3, and 4 h (Figure 7). The loss of *H. pylori* viability increased after 1-h incubation with AgNPs from 1.2 to 1.0 optical density, whereas *H. felis* shows a weak difference between treated and untreated; however, both strains showed a sharp decrease of growth from 1.0 to 0.6. By increasing the time of incubation, the loss of viability increased after 3 to 4 h of incubation. The division of cell death occurred in all 4 h of incubation; however, a large fraction of cell death occurred in the earlier hour of incubation. The treated groups show significant growth defect than control, whereas both control strains show health and no significant growth impairment.

### Anti-biofilm activity of AgNPs against *H. pylori*

**Figure 8:**
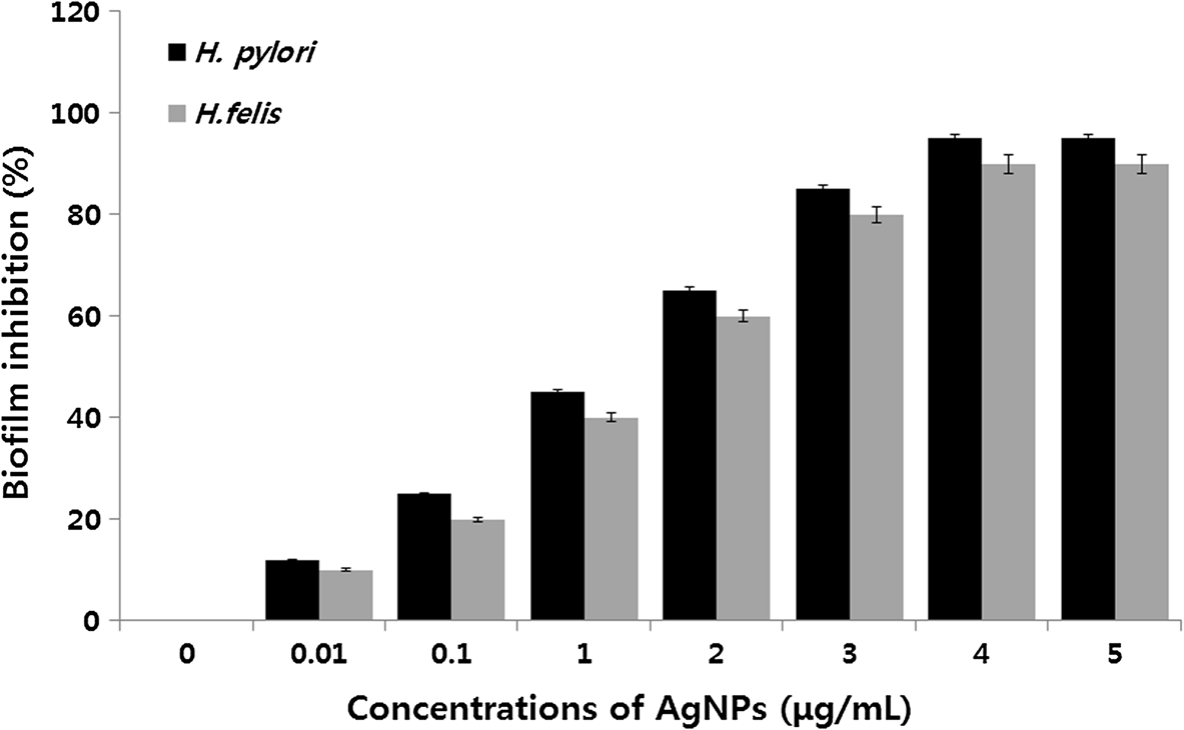
**Anti-biofilm activity of AgNPs.** The anti-biofilm activity of AgNPs was assessed by incubating all test strains with different concentrations of AgNPs for 24 h in a 96-well plate. The results are expressed as the mean ± SD of three separate experiments each of which contained three replicates. Treated groups showed statistically significant differences from the control group by Student’s *t*-test (*p* < 0.05).

Biofilms are surface-bound communities of microbial cells found in oligotrophic environments and are strongly implicated in bacterial virulence [55,85]. Yonezawa et al. [55] reported the biofilm activity of *H. pylori.* However, the anti-biofilm activity of AgNPs against *H. pylori* is recognized that it is still in its infancy. The purpose of the experiment was to design to investigate the inhibitory effects of AgNPs on biofilm formation by *H. pylori*. In this experiment, tissue culture plate was to assess the effect of AgNPs on inhibition of biofilm formation by *H. pylori*. Previously, we reported the antibacterial and anti-biofilm effects of AgNPs in *P. aeruginosa* [86]; therefore, this study was aimed to investigate the dose-dependent ability of AgNPs to inhibit the activity of biofilms by the human pathogen *H. pylori*, which is an unexplored microorganism. All test strains were grown for 24 h in microtiter plate wells and then treated with concentrations of AgNPs of 0.01 to 5 μg/mL (Figure 8). Treatment of *H. pylori* and *H. felis* for 24 h with 5 μg/mL of AgNPs shows decreased biofilm activity by more than 90 to 95%. Kalishwaralal et al. [86] reported that AgNPs inhibit biofilm formation against *P. aeruginosa* and *Staphylococcus epidermidis* using 100 nM of AgNPs which resulted in a 95 to 98% reduction in biofilm formation. Ansari et al. [87] demonstrated that AgNPs are able to inhibit production of exopolysaccharides, which are essential for biofilm formation. AgNPs also enhanced quorum quenching activity against *S. aureus* biofilm and prevention of biofilm formation [88]. Martinez-Gutierrez et al. [89] demonstrated that AgNPs could effectively prevent the formation of biofilms and kill bacteria in established biofilms. Taken together, our studies suggest that leaf extract *A. princeps*-mediated synthesis of AgNPs could be a viable anti-biofilm agent.

### Effect of AgNPs on ROS production

**Figure 9:**
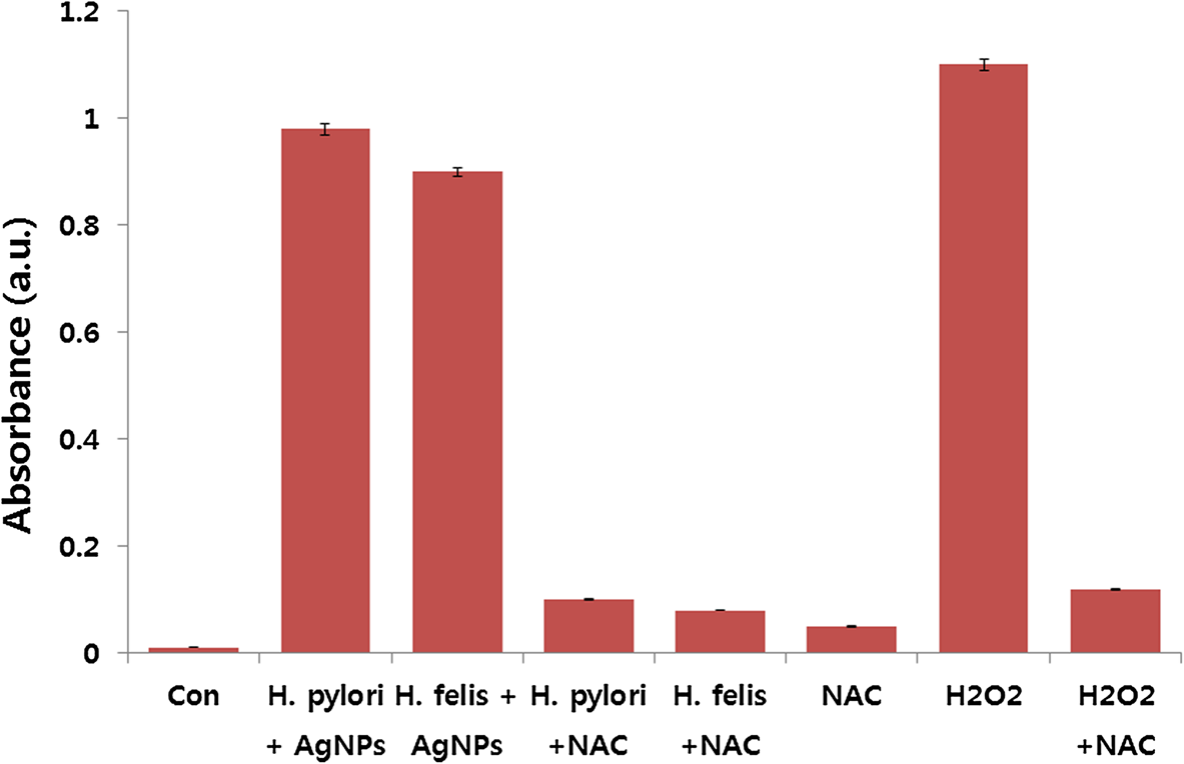
**Effect of AgNPs on the generation of ROS.** All test strains were treated with 1 μg/mL AgNPs for 12 h. ROS generation was measured by the XTT assay. The results are expressed as the means ± SD of three separate experiments, each of which contained three replicates. Treated groups showed statistically significant differences from the control group by Student’s *t*-test (*p* < 0.05).

To investigate the effect of AgNPs on ROS production, which is one of the key factors for bacterial cell death, *H. pylori* and *H. felis* were selected as a model bacterium to study the effect of ROS released from the surface of AgNPs on cell death. The bacterial cells were incubated with AgNPs for 12 h and then we measured the ROS production. The levels of ROS in AgNP- or H_2_O_2_-treated cells were higher, compared to the level of ROS in control cells [56]. H_2_O_2_ led to slightly higher ROS levels compared to the level of ROS in the AgNP-treated cells. Further, we tested if pre-incubation of cells with GSH or NAC could prevent ROS generation by AgNPs and found that these intracellular antioxidants protected *H. pylori* and *H. felis* from AgNPs and H_2_O_2_ (Figure 9). The data obtained from this study suggest that cell death is mediated by ROS production, which might alter the cellular redox status [56].

ROS is a natural by-product of the metabolism of respiring organisms [76]. Induction of ROS synthesis leads to the formation of highly reactive radicals that destroy the cells. The possible mechanisms of *H. pylori* and *H. felis* cell death are due to membrane damage by AgNPs which relates to metal depletion, that is, the formation of pits in the outer membrane and change in membrane permeability by the progressive release of lipopolysaccharide (LPS) molecules and membrane proteins [90]. Previous studies proposed that the sites of interaction for AgNPs and membrane cells might be due to sulfur-containing proteins present in the bacterial membrane proteins [91-95]. Previous studies suggest that ROS may be a common mechanism of cell death induced by bactericidal antibiotics [96-99].

### DNA fragmentation

**Figure 10:**
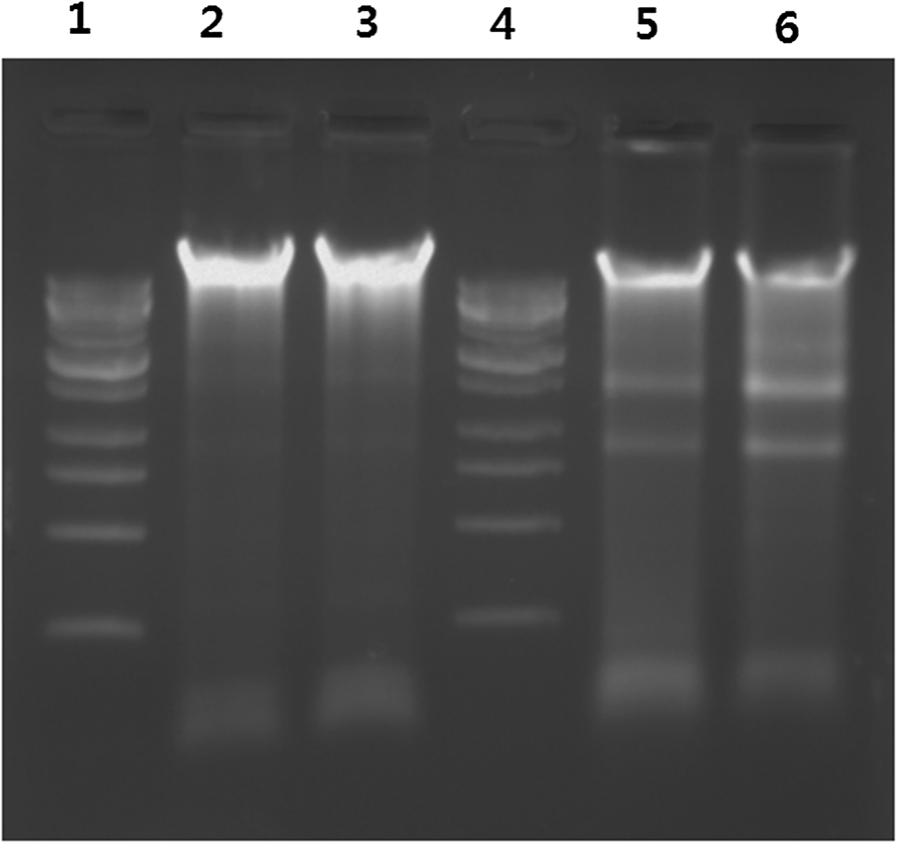
**Detection of AgNP-induced apoptosis by DNA fragmentation.** For DNA fragmentation analysis, cells were treated with 1 μg/mL for 12 h. After incubation, DNA extracted from cells and resolved on agarose gel electrophoresis. Lane 1, 1 kB ladder; lane 2, control from *H. pylori*; lane 3, control from *H. felis*; lane 4, 1 kB ladder; lane 5, AgNP-treated *H. pylori*; lane 6, AgNP-treated *H. felix.*

Apoptotic cell death is essential to the development and long-term viability of multicellular organisms [96,100,101]. Apoptosis can be induced in response to variety of intra and extracellular stimuli and stresses. A characteristic feature of apoptosis is the cumulative effects of biochemical and morphological changes which include cell-cycle arrest, halt DNA repair and homeostasis, inactivate apoptosis inhibitor proteins, and facilitate ultra-structural modifications, breakdown of cellular contents, and marking for death [96,102]. Previously, several studies shows that bactericidal antibiotics induces the generation of ROS, via a common metabolic mechanism, which contributes to drug-induced killing [98,103,104], which is due to production of ROS. Furthermore, Dwyer et al. [96] demonstrated that drug-induced bacterial cell death is indeed accompanied by DNA fragmentation, chromosomal condensation, and loss of structural integrity, all markers of eukaryotic apoptosis. We are interested to determine whether AgNPs could induce DNA fragmentation, which are physiological hallmarks of apoptosis in bacteria. To examine whether ROS generation by AgNPs leads to DNA damage in *H. pylori* and *H. felis*, DNA was extracted from both AgNP (1 μg/mL) treated cells with of AgNPs for 12 h and analyzed for the occurrence of DNA fragmentation. The results show that both strains treated with AgNPs for 12 h show laddering of DNA, specific DNA smearing is a characteristic feature of cell death [56], whereas untreated groups did not show significant fragmentation (Figure 10), which suggests that AgNPs are able to induce fragmentation of DNA free radicals produced by AgNPs. It is known that intracellular oxidative stress could be accelerated by NPs by disturbing the equilibrium between the oxidant and antioxidant processes [77]. ROS typically includes the superoxide radical (O^2−^), hydrogen peroxide (H_2_O_2_), and hydroxyl radical (OH), which cause damage to cellular components, including DNA and proteins [105,106]. ROS induces mitochondrial membrane permeability and damages the respiratory chain to trigger the apoptotic process [107]. The results drawn from DNA smearing studies indicate that AgNPs derived from *A. princeps* one of the potent inducer of ROS and eventually this ROS generation induces DNA fragmentation of cells. Although several studies have reported the antibacterial activity of biologically synthesized AgNPs in several microorganisms, this report describes that the maximum antibacterial and anti-biofilm effect with the lowest concentration of AgNPs in *H. pylori* and *H. felis.*

### Effect of AgNPs on cell viability of L132 and A549 cells

**Figure 11:**
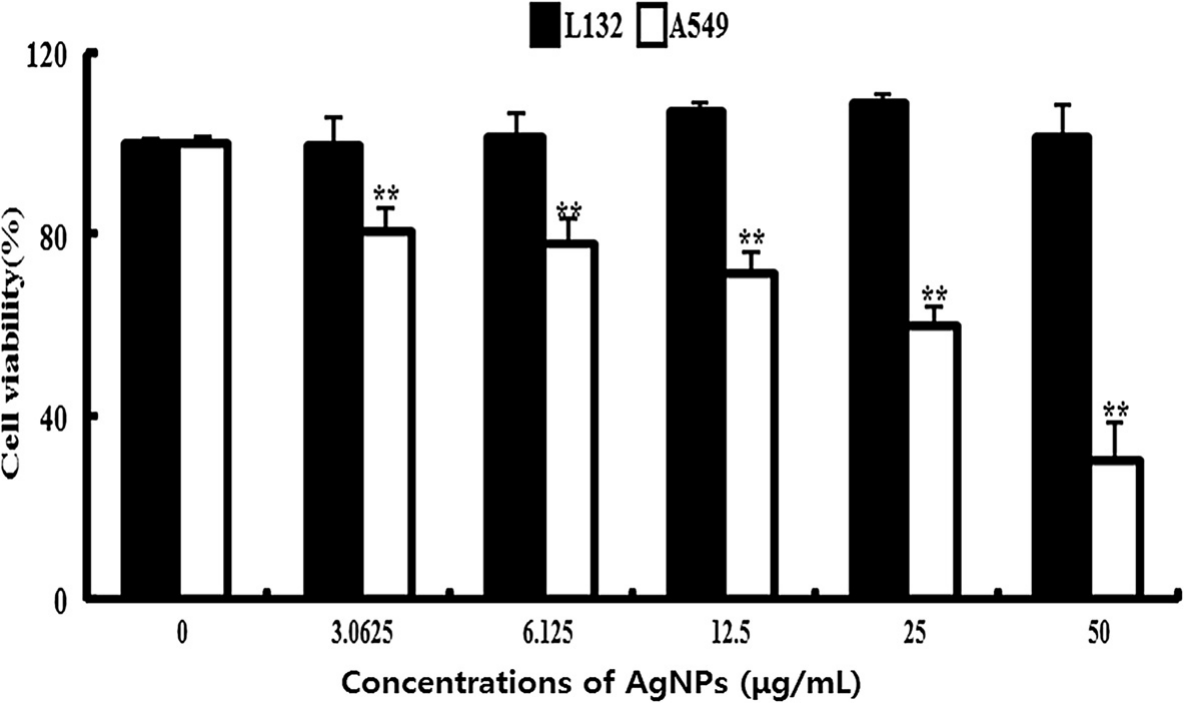
**Effect of AgNPs on cell viability of L 132 and A549 cells.** Cells were treated with various concentrations of AgNPs for 24 h, and cytotoxicity was determined by the MTT method. The results are expressed as the mean ± SD of three separate experiments each of which contained three replicates. Treated groups showed statistically significant differences from the control group by Student’s *t*-test (*p* < 0.05).

Although several studies showed the potential toxicity of AgNPs in cancer cells [51,54], to see the cell-specific activity of AgNPs, the effects of AgNPs on cell viability were evaluated using human lung cancer A549 cells and normal human lung L-132 cells; we selected A549 cancer cells and L-132 normal cells for our study because entry through the respiratory tract is one of the most frequent routes by which nanomaterials may enter the body. It was also to compare the cytotoxicity of nanoparticles in cancer cells and normal cells. Therefore, we are interested to investigate whether normal cells and cancer cells differentially respond to AgNPs, to examine the effect of both the cells which were treated with various concentrations of AgNPs and measured cell viability. After 24 h of treatment with different AgNP concentrations, the A549-treated cells showed a dose-dependent decrease in cell viability compared to that of the control group, whereas there is no significant difference between AgNP-treated L132 cells and control group (Figure 11). The most apparent and noticeable effect following exposure of cells to toxic materials is the alteration in the cell shape or morphology of the monolayer culture [108]. The cell viability assays shows that A549 cells have significant inhibitory effect of AgNPs and are more sensitive than L132 lung normal cells (Figure 11). Our studies are consistent with previous studies which show that AgNP exposure could induce the changes of cell shape, reduce cell viability, increase LDH release, and finally result in cell apoptosis and necrosis [48,51,53,109-111].

### Effect of AgNPs membrane leakage

**Figure 12:**
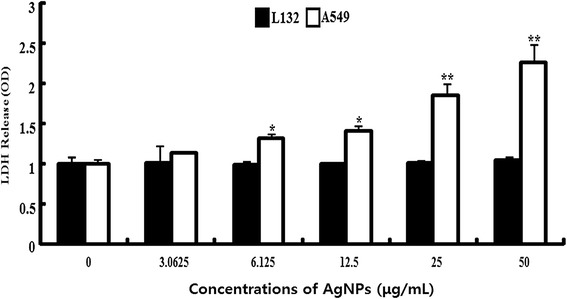
**Effect of AgNPs on LDH release from L 132 and A549 cells.** Lactate dehydrogenase (LDH) was measured by changes in optical density due to NAD^+^ reduction monitored at 490 nm, as described in materials and methods. The results are expressed as the mean ± SD of three separate experiments each of which contained three replicates. Treated groups showed statistically significant differences from the control group by Student’s *t*-test (*p* < 0.05).

To investigate the effect of AgNPs on membrane integrity; L132 and A549 cells were treated with various concentrations of AgNPs for 24 h. The results suggest that cell membrane leakage was dose dependent and significantly affected (Figure 12). The results from the LDH assay were consistent with cell viability; with increasing concentrations of AgNPs, the cells became gradually more cytotoxic. The increase of LDH leakage was due to abrupt cell membrane lysis following cell death, which suggests that the membrane leakage was a consequence of the apoptosis [54]. Interestingly, AgNP shows more toxicity in A549 cells, whereas L132 cells have no significant leakage of membrane. The results suggest that AgNP is targeting on cancer cells rather than normal in given concentrations. Similarly, Hussain et al. [41] observed that BRL 3A rat liver cells exposed to AgNPs for 24 h resulted in a concentration-dependent increase in LDH leakage and exhibited significant cytotoxicity at 10–50 μg/mL. Park et al. [112] reported that AgNPs significantly affect membrane integrity in L929 fibroblasts. Song et al. [113] observed dose- and time-dependent reduction of cell viability and decreased the activities of superoxide dismutase and glutathione peroxides. Lee et al. [114] reported that the level of LDH was increased to 210% when cells were exposed to 48 h in a culture medium containing AgNPs at 100 μg/mL. Interestingly, Choi et al. [115] found that exposure to layered metal hydroxide (LMH), for 72 h, resulted in a remarkable concentration-dependent increase in LDH leakage from A549 and HOS cells, but not from L-132 and HeLa cells, suggesting that the membrane damage caused by LMH depends on the cell types. Similarly, we found that membrane damage was observed only A549 cells. We found that concentration of 30 μg/mL is sufficient to induce 50% cell death, which is found to be IC50. Therefore, this concentration was used for further analysis of ROS and apoptosis assay.

### AgNPs induce time-dependent ROS generation

**Figure 13:**
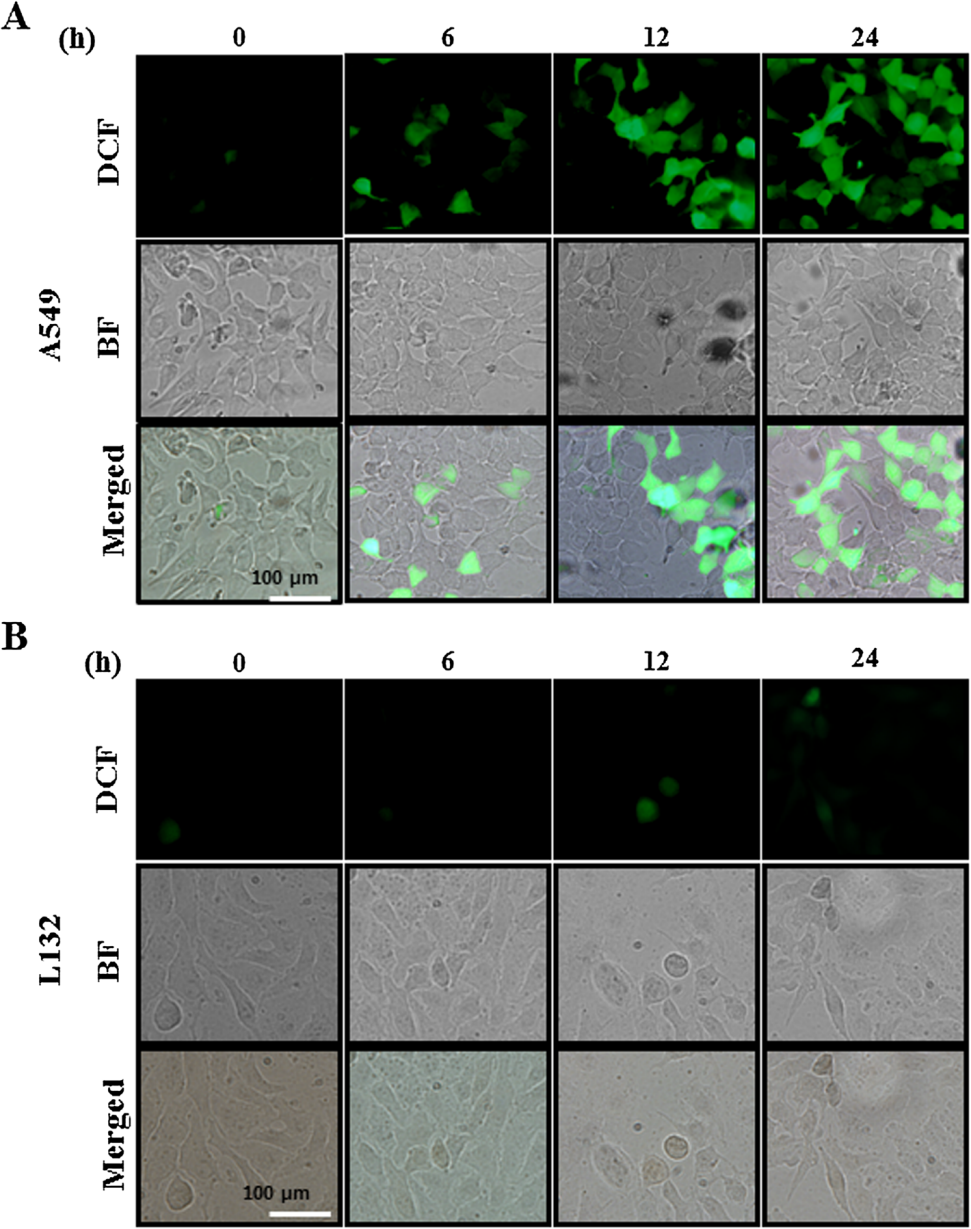
**AgNPs induce generation of ROS in AgNP-treated L 132 and A549 cells.** Fluorescence images of A549 **(A)** and L132 **(B)** cells without silver nanoparticles (AgNPs) (0) and cells treated with AgNPs 30 μg/mL and incubated at different time points. The image shows significant formation of hydrogen peroxide inside the A549 cells, whereas weak fluorescence was observed in L132 cells.

Since nanoparticles like carbon nanotubes [116], LMH [115], AgNPs [49,56], zinc oxide nanoparticles [117], graphene, and graphene-related nanomaterials [118] are toxic due to oxidative stress and lead to increased apoptosis. We examined the ability of AgNPs to induce oxidative stress by measuring ROS with carboxy-H_2_DCFDA in L132 and A549 cells. The cells were treated with 30 μg/mL of AgNPs at various time points such as 0, 6, 12, and 24 h. The increase in fluorescence intensity is directly proportional to the generation of ROS. A549 cells exposed to 30 μg/mL AgNPs for 24 h showed a dose-dependent increase in fluorescence intensity, indicating the generation of ROS and oxidative stress (Figure 13), whereas L-132 cells showed a very weak fluorescence at 24 h indicating AgNPs could target selective toxicity to cancer cells. Similarly, Choi et al. [115] found selective ROS generation by LMH in A549 cells. Akhtar et al. [117] also found that zinc oxide nanoparticles produce significant toxicity via ROS generation in all cancer cells including HepG2, A549, and BEAS-2B except normal rat cells (astrocytes and hepatocytes). In many pathological condition, inflammation-induced oxidative stress and ROS also plays an important role in lipid peroxidation followed by membrane damage [119].

### AgNPs induce mitochondrial-mediated apoptosis

**Figure 14:**
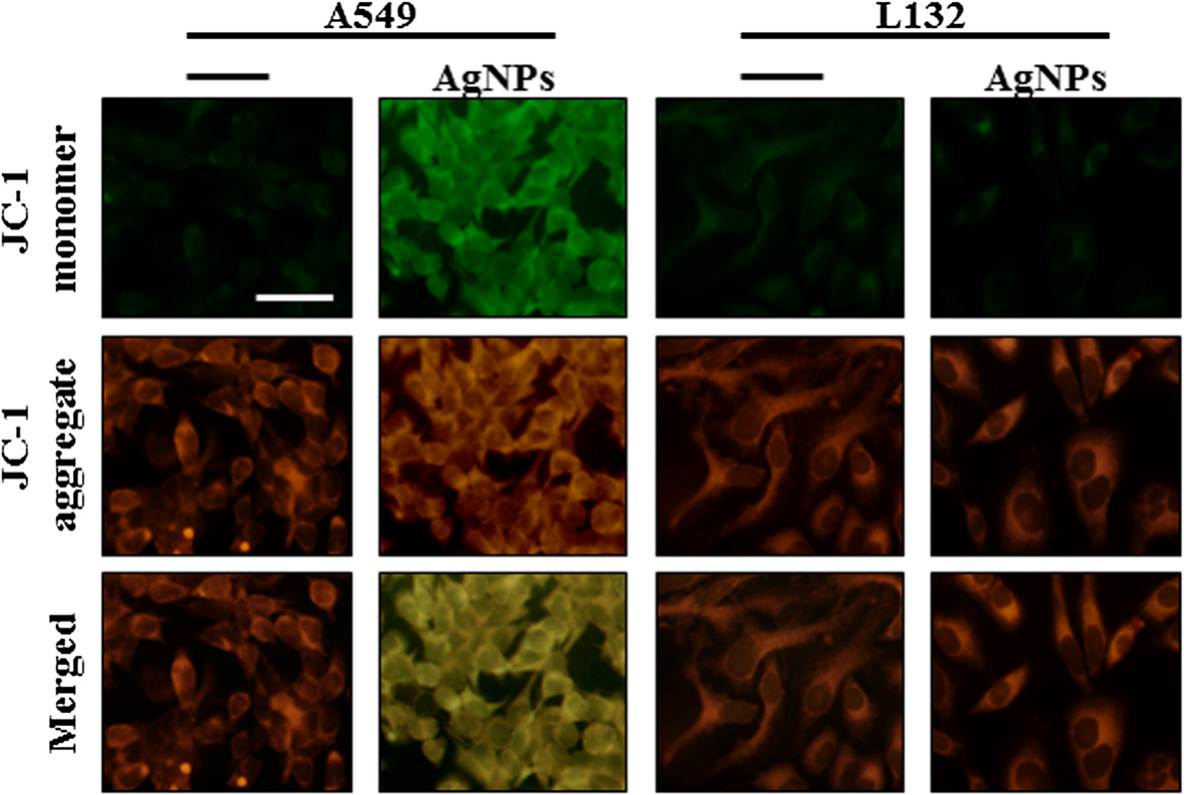
**AgNPs modulate mitochondrial transmembrane potential.** Changes in mitochondrial transmembrane potential (MTP) was determined using the cationic fluorescent indicator, JC-1. Fluorescence images of control A549 cells and L132 cells (without silver nanoparticles (AgNPs)) and cells treated with AgNPs (30 μg/ml).The changes of mitochondrial membrane potential by AgNPs were obtained using fluorescence microscopy. JC-1 formed red-fluorescent J-aggregates in healthy A549 cells with high MTP, whereas A549 cells exposed to AgNPs had low MTP and, JC-1 existed as a monomer, showing green fluorescence. L132 cells exposed to AgNPs shows healthy and had high MTP.

Mitochondrial transmembrane potential (MTP) is an early event in apoptosis. JC-1 monomer assay was used to evaluate the effect of AgNPs in mitochondrial membrane permeability. Mitochondria-mediated apoptosis undergoes two major changes which include changes in the permeabilization of the outer mitochondrial membrane and the loss of the electro chemical gradient [120]. The permeabilization of the outer membrane is tightly regulated by a member of the Bcl-2 family. Membrane depolarization is mediated by the mitochondrial permeability transition pore. Prolonged mitochondrial permeability transition pore opening leads to a compromised outer mitochondrial membrane [120,121]. As shown in Figure 14, decreases in mitochondrial energy transduction were observed following treatment of AgNPs for 12 h, illustrated by disappearance of red fluorescence and emergence of green fluorescence in A549 cells, whereas the green fluorescence was very weak in L132 cells treated with AgNPs at the same concentration and same time, indicating that AgNPs could cause MTP collapse significantly higher in cancer cells than normal cells. These results suggest that AgNPs could induce apoptosis through a mitochondria-mediated apoptosis pathway. A similar observation was made in RAW264.7 cells with the tert-butyl hydroperoxide treatment-enhanced mitochondria-mediated apoptosis through failure of MTP [122]. Mitochondria play an important role in apoptosis, via the intrinsic apoptotic program. An intrinsic apoptotic pathway is the depolarization of the mitochondrial (mt) membrane. Depolarized mt is a result of the formation of mt permeability transition (PT) pores [123,124]. mt PT has been associated with various metabolic consequences such as halted functioning of the electron transport chain with associated elevation in ROS and decreased production of ATP [125]. Govender et al. [123] observed a significant increase in mt depolarization after AgNP treatment, with an accompanying decrease in ATP concentration. They concluded that the high levels of bax expression, high mt depolarization, and decreased ATP suggest that AgNP induces cellular apoptosis in cancerous lung cells via the intrinsic apoptotic pathway. Several studies also suggest that nanoparticles seem to be localized in mitochondria and cause oxidative stress as well as potentiate structural damage and eventually lead to toxicity to the cells [44,126-128].

## Conclusion

We have demonstrated an easy, simple, and environmentally friendly approach to the synthesis of AgNPs using the leaf extract of *A. princeps* as a reducing and stabilizing agent. In this method, highly crystalline, spherical-shaped AgNPs with an average size of 20 nm were prepared without using any harmful reducing or capping agents. The phyto-molecules of the *A. princeps* extract were not only responsible for the reduction of AgNO_3_ but also function as capping agents to the surfaces of the AgNPs. The novel AgNPs show multifunctional effects against bacteria and human cancer cells, yet were biocompatible with normal lung cells. This suggests that AgNPs could contribute to develop therapeutic molecules for anticancer and anti-angiogenic. Interestingly, this comprehensive report describes the effect of AgNPs in bacteria and human cell types. Our results highlight a common and possible mechanism of cell death in bacteria and human cancer cells that is due to the generation of ROS, eventually leading to cell death. We believe that biologically prepared AgNPs could open a new avenue for various biomedical applications, particularly infections and cancer.
